# Variation in Four Horse Gait Categories Illustrated by Quantitative Analysis With ANALOC-E

**DOI:** 10.1155/vmi/4906015

**Published:** 2025-08-06

**Authors:** Elizabeth Ann Staiger, Adalton Pereira de Toledo, Victoria Rizzato Paschoal, Laura Patterson Rosa

**Affiliations:** ^1^Department of Animal Science & Veterinary Technology, Texas A&M University, Kingsville, Texas, USA; ^2^Toledo Horse, São Paulo, São Paulo, Brazil; ^3^Departamento de Nutrição e Produção Animal, Faculdade de Medicina Veterinária e Zootecnia, Universidade de Sao Paulo, Pirassununga, São Paulo, Brazil; ^4^Department of Veterinary Clinical Sciences, Lewyt College of Veterinary Medicine, Long Island University, Brookville, New York, USA

**Keywords:** gait, kinematic, marcha

## Abstract

Horse gaits are a trait highly selected and prized in diverse breeds. Meanwhile, gait classification relies mostly on subjective visual observations by evaluators. Noninvasive equipment able to track locomotion in horses and output quantitative gait parameters is not only helpful in evaluating locomotion but also in designating gait types and its variations. Equine locomotion pattern designation based solely on observer evaluation can be subjective; therefore, utilizing tools that provide quantitative results and track individual limb movements, especially during intermediate-speed gaits, can result in increased phenotypic accuracy and better designation. A noninvasive automated locomotion analysis system (ANALOC-E) was used to acquire locomotion parameters in a small yet diverse set of 68 horses of 4 breeds. We aimed to evaluate kinematic variation in horse locomotion patterns for these 68 horses. Analysis demonstrates kinematic variation within breed-designated gaits. We also compared output parameters to previously described standards, as well as assessed principal component scores within the dataset. We illustrate two gait types not described by the previous standards (marcha de centro and marcha trotada) yet recognized by breed designations. Three parameters (lateral support, diagonal support, and triple support) can explain about 98.9% of the variance in gait types in this dataset. Results suggest that ANALOC-E could be used for locomotion analysis, but further validation is necessary to evaluate the accuracy of the system. Noninvasive technologies that encourage natural locomotion and output quantitative biomechanical/kinematic parameters may assist in real-time, accurate, locomotion descriptions.

## 1. Introduction

Kinematic or biomechanical analysis evaluates the movement of body segments during locomotion, differentiating locomotion patterns by quantitative parameters. Its applications in quadruped species, especially in horses, range from clinical detection of locomotion abnormalities, such as lameness [1], to characterization of locomotion patterns [2, 3]. The Mangalarga Marchador (MM), Mangalarga (ML), and the Campolina (CA) are controlled by associations led by the Brazilian Department of Agriculture (MAPA), which sets strict rules for locomotion patterns and conformation standards. Only horses approved by a trained inspector can be fully registered and allowed for breeding and competition in the ridden classes at the breeds' shows [4, 5].

However, locomotion pattern analysis and designation solely based on observer evaluation can be subjective [6]. As the greatest variations in equine locomotion patterns occur at the intermediate speeds, which include the trot, pace, and ambling or four-beat gaits [2, 7, 8], kinematic evaluation utilizing tools that provide quantitative results and track individual limb movements is preferable [9]. These tools include inertial sensor/accelerometers attached to the horse [10, 11]. Yet, as horses possess increased dermal sensitivity and may be influenced by external forces originating from the equipment, invasive gear likely can interfere in the analysis outcomes and gait classification by introducing external stimuli that can alter the locomotion or its trajectory [12, 13]. Noninvasive automated methods such as the automated gait analysis through hues and areas (A.G.A.T.H.A.) and DeepLabCut can capture movement parameters using high-speed videos and machine learning [14, 15]. A system developed in the early 1990s to evaluate equid (Equus sp.) locomotion, named “Analisador de Locomoção – Equino” (ANALOC-E), outputs 21 kinematic parameters and an illustrated diagram of the locomotion pattern based on video analysis. This tool was developed mainly for the use in the MM, ML, and the CA horse breeds autochthonous to Brazil, which can perform distinct intermediate-speed locomotion patterns: the “marcha trotada” (MT), “marcha batida” (MB), and the “marcha picada” (MP) [4, 5, 16, 17].

We aimed to evaluate kinematic variation in a small yet diverse set of horse locomotion patterns of autochthonous Brazilian breeds. We evaluated 68 horses of the MM, ML, CA, and Criollo breeds with the ANALOC-E system, comparing output parameters to previously described standards [2]. We also assessed the discriminating kinematic parameters differentiating gait types within these breeds, and its quantitative variance and correlation associations to breed, sex, or speed. Our results demonstrate variation within breed-designated gait types including further quantitative description of two gaits, the MT and the marcha de centro (MC), and support the use and further research of automated, noninvasive locomotion analysis systems in the horse, especially for locomotion pattern quantitative classification.

## 2. Materials and Methods

### 2.1. Animals

Video sampling was authorized by the animal owners and conducted at different farms, where the animal was housed as part of a commercial service. Sampling at each farm was meant to keep the environment as familiar as possible to the horse and lessen possible environmental effects on locomotion. Each horse was previously evaluated for lameness and deemed fit to participate by each farm's resident veterinarian. All sampled animals were shown horses competing in their respective gait or performance classes and were under regular training with the farms' resident professional trainer or rider. Data were obtained for 68 horses representing 4 breeds, ranging in age from 4 to 12 years (mean = 5.87 and SD = 1.32), and consisting of 29 males and 39 females. Breeds included the ML (males = 6 and females = 11), MM (males = 8 and females = 20), CA (males = 12 and females = 8), and Criollo (*n* = 3, all males). The Criollos were included as an outgroup to represent a traditionally non-four-beat gaited breed from Brazil. All MM, ML, and CA individuals were fully registered with their respective breed associations (Associação Brasileira dos Criadores do Cavalo Mangalarga Marchador (ABCCMM), Associação Brasileira dos Criadores do Cavalo da Raça Mangalarga (ABCCRM), or the Associação Brasileira dos Criadores do Cavalo Campolina (ABCCC)). The three breed associations utilize the same standard and nomenclature for locomotion patterns (MB, MP, and MT), enabling joint and comparative analyses [4, 5, 16, 17].

### 2.2. ANALOC-E Sampling

**Figure 1:**
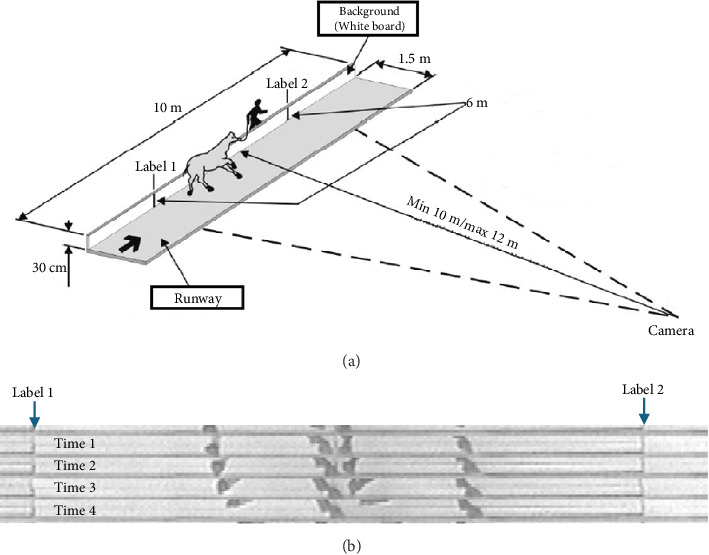
Graphical representation of the ANALOC-E system setup. (a) Demonstrating distances and positions of each of the system components and the subject. (b) Zoomed in view of the 6-m video capture area across 4 panels of consecutive (time) stance and swing detection in the ANALOC-E system.

Videos were captured, and locomotion was analyzed using the preassembled ANALOC-E system (available at https://www.toledohorse.com.br/analoc-e-analisador-de-andamentos-sem-contato-com-o-animal) between 1993 and 2005; locomotion reports were generated the same day after video capture. The following protocol was used, as recommended by the manufacturer: first, a runaway measuring 1.5 m wide and 10 m long was set up with a white board backdrop (30 cm wide and 10 m long) attached perpendicularly to the runway, which provides a neutral background to improve the identification of the horse's limbs. Two black-tape markers were placed 6 m apart on the background (Label 1 and Label 2) to delimit the video capture area. A Panasonic AG-DVC60 (Panasonic Brasil, Sao Paulo, SP) camera was placed on a tripod located between 10 and 12 m away (depending on farm location where samples were collected) from the background and positioned 30 cm above the ground (Figure 1(a)). A resident professional rider or trainer of average 65 kg (143 lbs) body weight at each farm rode the subject animal at the animal's favored intermediate gait and speed, utilizing the individual animal's preferred riding and training equipment (i.e., saddle, bridle, and bit) to minimize variation due to novel equipment, rider, or riding style. The camera was set to record using the “regular shooting” mode per the manufacturer's recommendations, with automatic shutter speed, iris, white balance, gain, and focus, a 440,000 effective pixel resolution, and a frame rate of 60 frames per second, and using an optical image stabilizer lens set to f/1.6 aperture. The camera was connected to a computer (micro-PC Pentium with Windows XP, 128 Mb RAM and 2.4 Gb HD) using a digital video (DV) cable.

**Figure 2:**
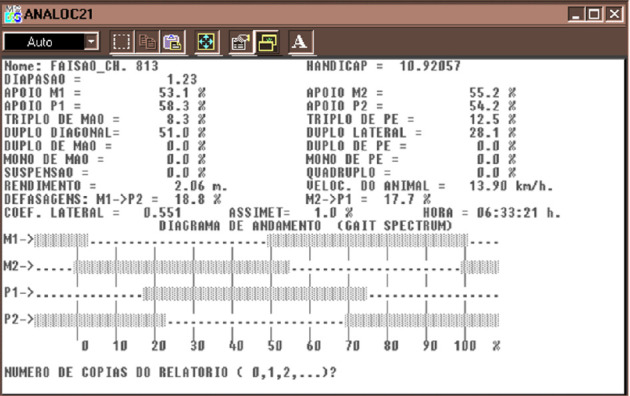
Graphical ANALOC-E output example of a Mangalarga Marchador horse. The upper section demonstrates in percentages each of the kinematic parameters evaluated. The locomotion pattern diagram (gait spectrum) is a graphical representation of the locomotion pattern, where M1 and M2 denote the forelimbs and P1 and P2 represent the hind limbs. For each limb, the continuous line represents the stance phases, and the dotted line represents the swing phases. Translations: TRIPLO DE MAO = triple support with single forelimb; DUPLO DIAGONAL = diagonal support; DUPLO DE MAO = double support fore; MONO DE MAO = single support fore; SUSPENSAO = suspension; TRIPLO DE PE = triple support with single hind limb; DUPLO LATERAL = lateral support; DUPLO DE PE = double support hind; MONO DE PE = single support hind; QUADRUPLO = quadrupedal support; VELOC. DO ANIMAL = speed (km/hr).

The ANALOC-E system recognizes and evaluates image frames below the carpal and tarsal joints on each subject, being composed of three software platforms: TT-EGME2, Pocotó 40 (PCT40), and Diapasão 21 (DIAP21). During sample collection, the images are processed using the main ANALOC-E executable software. First, the operator uses the “rectangle” delimiter in the software to reduce the field of capture zone down to the 6-m video capture area that contains stance and swing phases of each limb for at least three complete strides. Images are then processed first by TT-EGME2 for image acquisition, and subsequently by PCT-40, which improves the image frame rate and quality (Figure 1(b)). PCT-40 also defines the times of stance and swing phases of each limb and analyzes limb support type (monopedal, bipedal, tripedal, and quadrupedal) per stride. Lastly, DIAP21 combines the quantitative kinematic parameters into graphical data and analytical representations, generating final reports as an executable file. The results are combined in a visual output by ANALOC-E software, with the individual locomotion parameters in percentages of the full locomotion analysis, as well as a graphical locomotion pattern diagram called the gait spectrum (Figure 2).

### 2.3. ANALOC-E Kinematic Parameter Outputs

For the 68 horses, a minimum of five strides were utilized to evaluate the locomotion parameters. Despite 21 parameters totally being generated, only 12 were consistently preserved for all 68 records. We selected 11 parameters (Table S1) for further analysis and characterization of locomotion pattern performed by each individual horse. As measured by the ANALOC-E equipment, the total tripedal support (TS) is composed of pelvic TS (TS_P; supported by 2 hind limbs and 1 fore) and thoracic TS (TS_T; supported by 2 fore limbs and 1 hind). Measurements not utilized in our analysis due to lacking preserved data or source for the mathematical model utilized were the bipedal coefficient, dissociation, and diapason. The bipedal coefficient is an automatic ratio calculated by the ANALOC-E system corresponding to a predominance of lateral stances (values above 1) or predominance of diagonal stances (values below 1). Dissociation, illustrated in the gait spectrum (Figure 2) as M1>P2 and M2>P1, refers to the time between support stance of the forelimb (M1 or M2) and support stance of the respective diagonal hind limb (P1 or P2). Diapason or “Diapasão,” the only parameter conserved in all samples yet lacking manufacturer information on the respective mathematical model used to achieve the output result, is a second automatic ratio corresponding to a relationship between support and swing time of the four limbs during one stride, where, in the four-beat gaits, it is equal or greater than 1 and, in two-beat diagonal gaits, it is less than 1.

### 2.4. Statistical Analysis

We classified locomotion pattern type two ways: (1) as reported by the breed registry for each individual horse and (2) by comparing the lateral support (LS), diagonal support (DS), single, quadrupedal, and tripedal support outputs to average, maximum, and minimum values of previously published criteria [2] (Table S1). Statistical analysis was performed using JMP Pro 15 (SAS Institute, Inc, Cary, NC). An Anderson–Darling test was used to test for normality in the parameters. Speed, TS, and stride length measures are normally distributed across the breeds. Using nine of the quantitative stance and suspension measures, we performed a principal component analysis (PCA) to create covariance-matrix principal component (PCv) values for each individual. The PCv values were plotted and labeled based on breed standards and previously published gait-type classifications to examine how the individuals cluster according to the classification method. We performed a second PCA based on a correlation matrix using all 11 quantitative measures. The correlation principal component (PCr) values were plotted and labeled based on breed standards gait-type classifications to examine within breed variation. Due to the non-normal distribution, we evaluated possible effects of sex and breed compared to PCv1, PCv2, PCr1, and PCr2 using a Wilcoxon or Kruskal–Wallis nonparametric test. Results were considered statistically significant at a Bonferroni corrected value of *p* ≤ 0.01.

We analyzed differences in PCv scores due to age, speed, and stride length and differences in PCr scores due to age using Pearson and Spearman's pairwise correlations. Results were considered statistically significant at *p* < 0.05. To assess the impact of including speed and stride length in the PC calculation interpretation for within breed variation, we compared the effect of breed on speed and stride length using an ANOVA test. We used the post hoc Tukey–Kramer HSD test to identify which means significantly differed, accounting for multiple comparisons.

## 3. Results

The 68 equids displayed variation across the kinematic measures. Means and standard deviations across the sample set are presented in Table 1.

### 3.1. Principal Component Results

**Figure 3:**
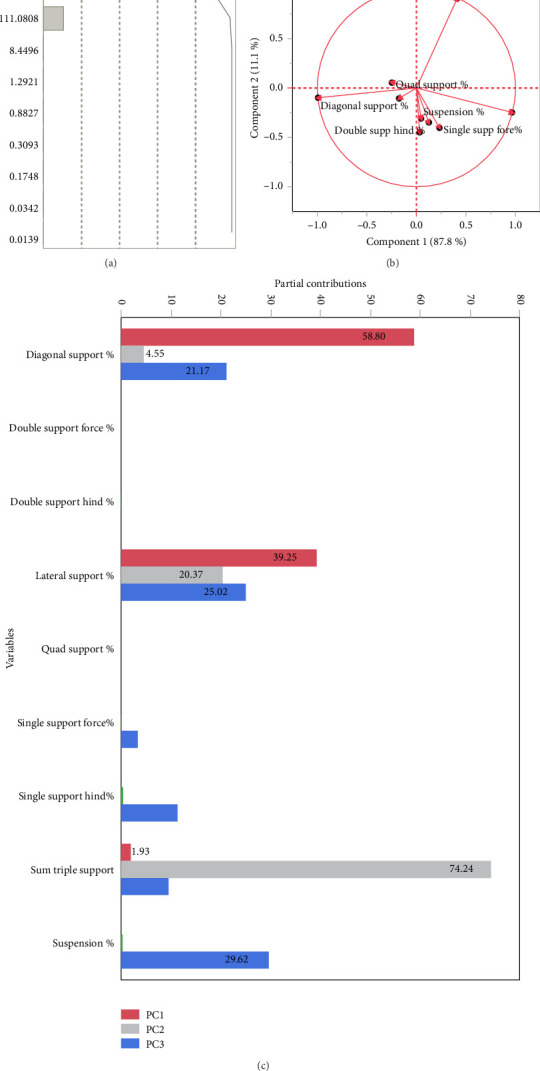
Covariance principal component (PCv) analysis of the 9 stance and suspension parameters for 68 horses. (a) The eigenvalues and percent explained used to determine the number of components retained for further analysis. (b) Component loading and (c) partial contribution of variables plots used to interpret the PCvs.

Based on the eigenvalues and percent explained (Figure 3(a)), we retained PCv1 and PCv2 for further analysis in 68 horses. PCv1 explains 87.8% of the variance and PCv2 explains about 11.1% of the variance, totaling 98.9% of the variance observed. For PCv1, the major contributors included DS, which loads in a negative direction, and LS, which loads in a positive direction. Double support fore and quadrupedal support also load in a negative direction, while the remaining supports and suspension load in a positive direction. For PCv2, the major contributors included TS, which loads in a positive direction, and LS, which loads in a negative direction (Figures 3(b) and 3(c)).

**Figure 4:**
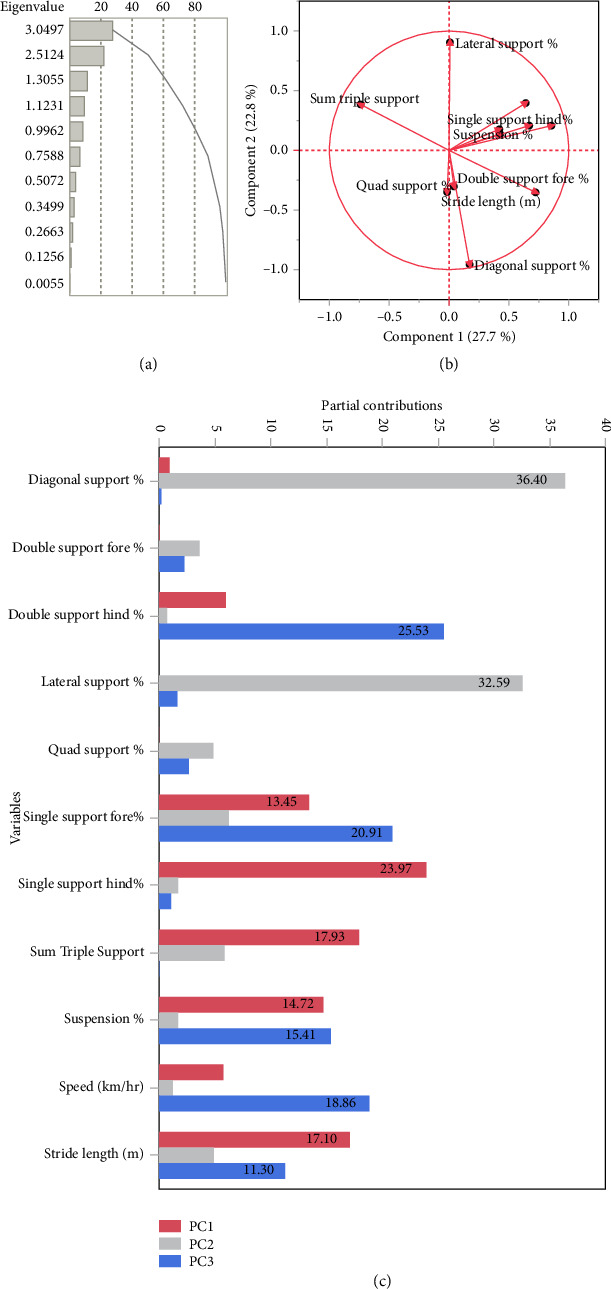
Correlation principal component (PCr) analysis of 11 quantitative gait parameters for 68 horses. (a) The eigenvalues and percent explained used to determine the number of components retained for further analysis. (b) Component loading and (c) partial contribution of variables plots used to interpret the PCrs.

Using the eigenvalues and percent explained, we retained PCr1 and PCr2 for further analysis. PCr1 explains 27.7% of the total variation, and PCr2 explains 22.8% of the variation. The major contributors to PCr1 include single support hind and stride length, which load in a positive direction, and TS which loads in a negative direction. PCr2 is described by DS, loading in a negative direction, and LS which loads in a positive direction (Figure 4).

### 3.2. Gait Classification

According to breed registry parameters, 35 individuals were classified as MB, seven as MP, 16 as MT, two as broken trot, one as broken pace, and five as unclassified. Two individuals could not be classified in either MB or MP but performed a unique pattern that spent an equal amount of time in LS and DS, intercalated by TS stances, popularly recognized in Brazil as “MC” (center gait, MC) [16, 18]. Based on the previously described gait-type parameters [2], 30 individuals could not be classified, while 14 were classified as “Rocky Mountain Rack,” 13 as “Foxtrot,” three as “Coon Rack,” two as “Tölt,” two as broken trot, one as MB, one as “Sobreandando,” one as “Paso Llano,” and one as “Curly Rack.” Breed registry and the previously described gait-type parameters [2] only agreed for gait classification in five individuals, two of which demonstrated a “broken” or uncoupled trot, and three of which were uncharacterized for both classifications.

**Figure 5:**
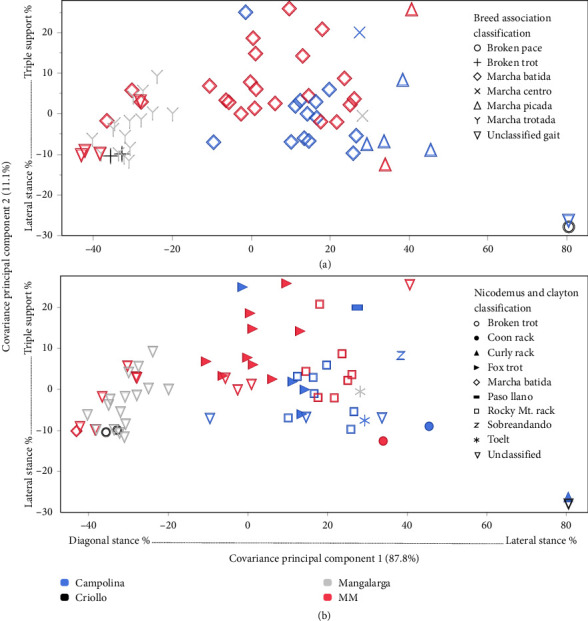
Comparison of PCv1 scores to PCv2 scores. Colors represent breed, and shapes represent the gait type based on (a) breed registry standards or (b) previously described gait parameters [2].

Plotting the PCv scores based on the gait classifications identified clusters of horses that perform predominately diagonal gaits, a mix of lateral and diagonal gaits with TS, and LS gaits (Figure 5). In reference to the breed registry standards, four MM horses classified as MB clustered with ML horses classified as MT. The two MC horses separate the MP cluster from the MB cluster. Of the five unclassified horses, one clustered with “broken pace,” one with “MT,” and three with “broken trot.”

### 3.3. Breed Associations to PC Scores and Parameters

Horse breeds demonstrated a significant association to both PCv1 (*X*^2^ (3, *N* = 68) = 25.98, *p*=9.622 × 10^−6^) and PCv2 (*X*^2^ (3, *N* = 68) = 14.99, *p*=0.0018). This relationship revealed that the ML spends more time in DS versus LS, and the Criollo shows virtually no TS stances (Figure 5). Sex was not significantly associated with PCv1 (*X*^2^ (1, *N* = 68) = 2.70, *p*=0.1004) or PCv2 (*X*^2^ (1, *N* = 68) = 0.79, *p*=0.3753).

**Figure 6:**
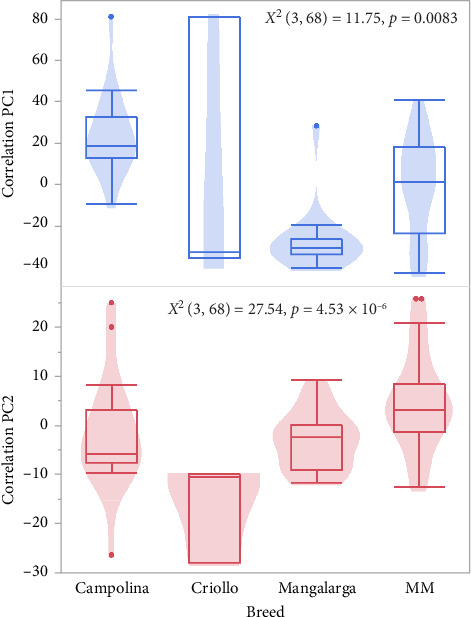
Violin plot of correlation principal component (PCr) 1 and PCr2 scores demonstrating breed-specific variation.

Breed also had a significant association with PCr1 (*X*^2^ (3, *N* = 68) = 11.76, *p*=0.0083) and PCr2 (*X*^2^ (3, *N* = 68) = 27.54, *p*=4.53 × 10^−6^). This relationship indicates that Criollo spends more time in single-support phases and less time in TS, which allows for a longer stride length, and that ML spends more time in DS (Figure 6). Sex was not significantly associated with PCr1 (*X*^2^ (1, *N* = 68) = 1.15, *p*=0.2834), but it was associated with PCr2 (*X*^2^ (1, *N* = 68) = 5.522, *p*=0.0188), where males had a higher PCr2 score than females, suggesting that males likely spend more time in LSs, while females prefer diagonal stances. Yet, this result can be due to the confounding effect of more females represented in predominantly diagonal gaits like the MB and MT, while males had more representatives in a predominantly lateral gait, MP.

**Figure 7:**
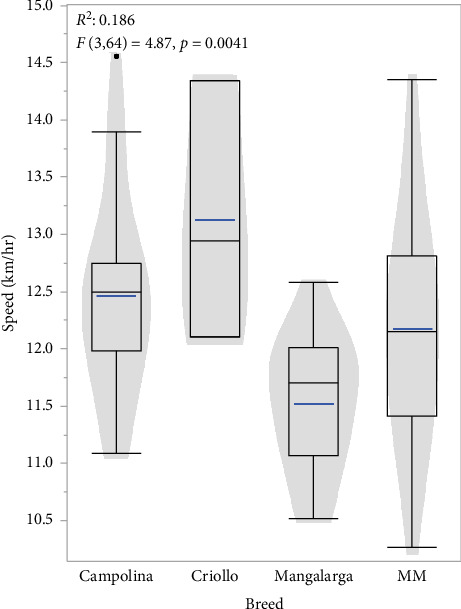
Violin plot comparison of speed across breeds, demonstrating the speed parameter distribution for the Criollo, MM, ML, and CA horses.

When comparing the effect of breed on speed and stride length, there was a significant association between speed and breed [*F*(3, 64) = 4.87, *p*=0.0041] suggesting that Criollo horses are on average faster than other analyzed breeds when performing their preferred gait (Figure 7) and indicating a possible confounding effect of speed on the PCs due to the intrinsic locomotion patterns demonstrated by the individuals sampled. There was no significant association between stride length and breed [*F* (3, 64) = 2.28, *p*=0.0878].

## 4. Discussion

Noninvasive methods able to quantitatively differentiate equine gait kinematic variations are critical for lameness detection and selection programs. We evaluated the quantitative locomotion outputs to discriminate kinematic parameters for different four-beat gait types, as well as compared with previously described standards for gaited horses [2]. Our findings indicate that there is greater variation in the kinematic parameters than previously reported especially regarding these four Brazilian autochthonous breeds. Further validation is required in a larger dataset and in other breeds demonstrating diverse locomotion patterns to compare the accuracy of the used system with gold-standard practices and wearable technology and to reproduce the variation demonstrated in this dataset. It is possible that part of the reported variability and overlap in parameters can be generated by measurement, computational, or technological errors in the system. The potential benefit of the noninvasive systems would be in the evaluation of young horses not yet acclimated to the attachment of equipment, such as splint boots, girths, and/or tape, and the use of the background board to neutralize the confounding effects of background or the handler's legs on the identification of the horse's limbs.

Equine gaits at intermediate speeds have been previously described as forming a continuum, with named gaits merging into one another [2, 3, 19]. However, discrete gait classification is essential for competition horses to ensure that similar gait patterns are compared. Therefore, providing baseline kinematic parameters to characterize the differences in gaits across breeds was established by Nicodemus and Clayton [2]. We used these baseline categories to broadly classify the horses' gaits due to their inclusion of MB and MP in their analysis. Our results support the kinematic continuum of gait types, but also that the baseline categorization needs to be updated. For example, several MM horses classified by their breed registry as performing the “MB,” we classified as either performing the “Fox Trot” or the “Rocky Mountain Rack” based on the Nicodemus and Clayton [2] parameters. These two groups did form separate clusters in our PCA analysis, but they did not cluster with MB; the only horse classified as MB is clustered with the “broken trot” classification, and the breed registry had not classified the horse as MB. While Nicodemus and Clayton [2] did utilize horses defined as the best representatives of each gait from the breed registrations, a major limitation was the small sample size that may not have captured the true variation observed among performers of the breed and gait. Furthermore, not all the possible gait classifications were included in their analysis, specifically “MT.”

To define equine gaits, stance durations, footfall, and timing sequences are typically used. Linear discriminant analysis or PCA can be used on the kinematic parameters to improve the classification process [3, 20, 21]. PCA was applied since classification of the gaits was performed within a similar speed spectrum. The PCA successfully distinguished gaits based on percentages of DS, LS, and TS under a covariance matrix. Under a correlation matrix, stride length and single support further defined the gaits. The higher average values for DS on MT and MB and for LS on MP and MC were corroborated by the breed registry gait definitions for MT and MB as mostly diagonally coupled gaits, while MP is mostly a laterally coupled gait [2, 14, 17]. As demonstrated by the variance explained in PCv1, DS and LS seem to have the strongest effect on different gait types even between diverse breeds. A qualified inspector (veterinary or animal science professional) policed and licensed by the breed association, and MAPA must perform two inspections for each individual prior to registration in the breed's studbook. The first inspection is performed before 2 years of age for parentage via DNA and overall health, resulting in a provisory registration. The second inspection evaluates gait and conformation when the horse is under saddle, usually around 2-3 years of age, and includes a riding evaluation by the inspector. All horses sampled in this study were actively competing in their respective breed-assigned gait classes. Due to the strict selection for gait of the MM, ML, and CA, where they are judged for both registration and competition by 3–5 specialized judges, these breeds can be reliable models for locomotion pattern studies.

The relationship of the parameters described by PCr1 indicates that horses spending more time in single-support phases will have a longer stride length and shorter TS phase. During the gait cycle, the decrease in a parameter percentage will likely incur an increase in a different parameter; thus, the association of increased single support decreasing TSs is not surprising. Yet, the observation in longer stride lengths requires further investigation. Unfortunately, the association between these parameters could be an effect of the small sampling in this study. Incorporating other trotting and broken trot breeds, along with individual morphometric measurements, would aid in teasing out the association with stride length. Surprisingly, the Criollo breed had higher PCr1 values compared to the other breeds; we would have expected the CA breed to have the highest scores due to their reported morphometrics [22, 23]. While morphometric variations have been demonstrated to not directly impact the gait-type [24], they may still have a role in the quality of the locomotion performance [25, 26]. A longer scapula is associated with longer strides [25], and while we did observe differences in stride length by breed (Criollo had the longest, followed by CA, ML, and MM), they were not statistically significant. A potential confounder could be the small sample size of the Criollo breed, where we would likely observe a different stride length average with a larger sampling. We do not have available morphometric measurements of the sampled horses to further test the impact of morphological variation on the gait parameters.

Two individuals demonstrated a locomotion pattern with parameters between MB and MP. Although its kinematics are not previously described, this gait type is recognized in Brazil as “MC” [16, 18]. However, the distribution of parameters for these two individuals also resembles three of the 16 intermediate-speed four-beat gaits described by Nicodemus and Clayton [2]: classic fino, paso llano, and tölt. The variability of locomotion parameters in four-beat intermediate gaits and overlapping of these, as observed in our cohort, demonstrates that the gait-type classification can benefit from the observation of more parameters beyond footfall, stances, and timing sequences, as well as nonridden observations. A noninvasive approach using artificial intelligence DV analysis was able to detect subtle changes in equine gait in response to fatigue, associated with multiple parameters beyond the gait diagram [27]. Furthermore, future analysis could benefit from the incorporation of nonlinear dynamic measures, such as the Lyapunov exponent, to quantify the stability and adaptability of equine gait. This metric, which has been used in human gait analysis to identify chaotic behavior and step-to-step variability, could provide valuable insights into locomotor control in horses and help distinguish between physiological variation and pathology [28].

## 5. Conclusions

We demonstrate variation in quantitative measurements of horse locomotion patterns using a noninvasive, video-based technology for gait analysis that can improve and expand on locomotion analysis and classifications for these four Brazilian autochthonous breeds. Three parameters (LS, DS, and TS) represented by PCv1 and PCv2 can explain about 98.9% of the variance in gait types of Brazilian gaited breeds. However, the ANALOC-E system and other noninvasive systems do warrant validation testing from a broader sampling pool representative of additional gait types, age, and soundness classifications. Noninvasive technologies that encourage natural locomotion and output quantitative biomechanical/kinematic parameters may assist in real-time accurate locomotion descriptions, assessment of quality, and pathologies of the lame horse's locomotion.

## Figures and Tables

**Table 1:** Mean (±S.D.) measures for traits of interest in 68 equids.

Traits	CA (*n* = 20)	Criollo (*n* = 3)	MM (*n* = 28)	ML (*n* = 17)
Age (years)	5.95 (±1.57)	6.67 (±1.15)	6.536 (±2.06)	5.353 (±1.0)
Diagonal support (%)	43.76 (±13.93)	56.77 (±49.19)	60.371 (±19.61)	82.594 (±11.94)
Double support fore (%)	0 (±0)	0 (±0)	0.036 (±0.19)	0 (±0)
Double support hind (%)	0.09 (±0.40)	0 (±0)	0.068 (±0.25)	0 (±0)
Lateral support (%)	37.55 (±15.52)	26.3 (±45.55)	18.868 (±13.67)	5.747 (±9.24)
Quadrupedal support (%)	0 (±0)	0 (±0)	0.032 (±0.17)	0.382 (±0.81)
Single support fore (%)	0.045 (±0.20)	3.13 (±3.20)	0 (±0)	0.112 (±0.46)
Single support hind (%)	0.26 (±1.18)	5.13 (±1.09)	0.30 (±1.11)	0.229 (±0.95)
Triple support (%)	18.28 (±8.16)	0 (±0)	20.157 (±10.16)	9.682 (±6.32)
Suspension (%)	0 (±0)	8.63 (±4.37)	0 (±0)	0 (±0)
Speed (km/hr)	12.46 (±0.89)	13.13 (±1.13)	12.172 (±1.0)	11.523 (±0.59)
Stride length (m)	1.97 (±0.13)	2.17 (±0.11)	1.927 (±1.19)	1.97 (±0.14)

## Data Availability

All raw data outputs supporting the reported results are freely available and can be found in the supporting Table S1.

## Supporting Information

Table S1: Individual, Kinematic, and Resulting Principal Component Values of the 68 analyzed horses.
