# Supplementary material for: Variation in Four Horse Gait Categories Illustrated by Quantitative Analysis With ANALOC-E
Source: Vet Med Int. 2025 Aug 6;2025:4906015. doi: 10.1155/vmi/4906015 (PMC12349990; doi:10.1155/vmi/4906015)
Supplement: Supporting Information — Additional supporting information can be found online in the Supporting Information section. [file 4906015.f1.docx]

| Supplemental Table 1. Individual, Kinematic and Resulting Principal Component Values of the 68 analyzed horses. | | | | | | | | | | | | | | | | | | | | | | | | | |
| --- | --- | --- | --- | --- | --- | --- | --- | --- | --- | --- | --- | --- | --- | --- | --- | --- | --- | --- | --- | --- | --- | --- | --- | --- | --- |
| Horse ID | Age | Breed | Nicodemus and Clayton Classification [2] | Breed Registry Classification | Sex | Diagonal Support % | Double Supp Fore % | Double Supp Hind % | Lateral Support % | Quad Support % | Single Supp Fore% | Single Supp Hind% | Sum Triple Support | Suspension % | Diapasao | Speed (km/hr) | Stride Length (m) | Total Double Supp% | Total Single Supp% | PCr1 | PCr2 | PCr3 | PCv1 | PCv2 | PCv3 |
| ANALOC-E_36 | 5 | Campolina | Rocky Mt. rack | Marcha Batida | F | 44.6 | 0 | 0 | 31.3 | 0 | 0 | 0 | 24.1 | 0 | 1.27 | 11.18 | 1.93 | 75.9 | 0 | -1.08847 | 0.789922 | -0.25354 | 19.7795 | 5.957506 | 0.334332 |
| ANALOC-E_12 | 5 | Campolina | Unclassified | Marcha Batida | F | 52.2 | 0 | 0 | 34.8 | 0 | 0 | 0 | 13.1 | 0 | 1.14 | 13.75 | 1.95 | 87 | 0 | 0.10478 | 0.697239 | 0.979963 | 14.61495 | -6.72079 | 2.190445 |
| ANALOC-E_21 | 5 | Campolina | Rocky Mt. rack | Marcha Batida | F | 48.1 | 0 | 0 | 30.8 | 0 | 0 | 0 | 21.2 | 0 | 1.24 | 12.52 | 2.01 | 78.9 | 0 | -0.40984 | 0.657594 | 0.514751 | 16.37907 | 2.938122 | 0.800454 |
| ANALOC-E_46 | 4 | Campolina | Rocky Mt. rack | Marcha Batida | F | 55.7 | 0 | 0 | 32.1 | 0 | 0 | 0 | 12.3 | 0 | 1.13 | 12.48 | 2.04 | 87.8 | 0 | 0.066253 | 0.234153 | 0.569848 | 10.12813 | -6.93777 | 2.203515 |
| ANALOC-E_43 | 5 | Campolina | Fox Trot | Marcha Batida | F | 52.3 | 0 | 0 | 28 | 0 | 0 | 0 | 19.6 | 0 | 1.22 | 12.56 | 2.07 | 80.3 | 0 | -0.16059 | 0.346886 | 0.633907 | 11.18158 | 1.927715 | 0.838917 |
| ANALOC-E_27 | 5 | Campolina | Paso llano | Marcha Centro | F | 34.7 | 0 | 0 | 28.7 | 0 | 0 | 0 | 36.6 | 0 | 1.45 | 11.36 | 1.77 | 63.4 | 0 | -2.02582 | 1.518019 | -0.52793 | 27.48022 | 20.01248 | -1.66733 |
| ANALOC-E_63 | 5 | Campolina | Toelt | Marcha Picada | F | 41.1 | 0 | 0 | 44.4 | 0 | 0 | 0 | 14.4 | 0 | 1.16 | 14.55 | 2.02 | 85.5 | 0 | 0.384398 | 1.302761 | 1.57266 | 29.32238 | -7.56676 | 2.286373 |
| ANALOC-E_53 | 10 | Campolina | Curly Rack | Unclassified Gait | F | 4.4 | 0 | 1.8 | 83.3 | 0 | 0.9 | 5.3 | 4.4 | 0 | 0.98 | 12.66 | 2.23 | 89.5 | 6.2 | 4.502463 | 3.976539 | 4.050986 | 80.46411 | -26.2936 | -0.1717 |
| ANALOC-E_9 | 8 | Campolina | Fox Trot | Marcha Batida | M | 50.5 | 0 | 0 | 30.9 | 0 | 0 | 0 | 18.4 | 0 | 1.2 | 12.11 | 1.84 | 81.4 | 0 | -0.82068 | 0.716174 | -0.02459 | 14.212 | -0.03135 | 1.091432 |
| ANALOC-E_62 | 8 | Campolina | Unclassified | Marcha Batida | M | 70.8 | 0 | 0 | 19.8 | 0 | 0 | 0 | 9.5 | 0 | 1.1 | 11.09 | 2.19 | 90.6 | 0 | 0.28417 | -0.96493 | 0.146497 | -9.54688 | -7.01853 | 2.134884 |
| ANALOC-E_51 | 6 | Campolina | Rocky Mt. rack | Marcha Batida | M | 42.6 | 0 | 0 | 41.6 | 0 | 0 | 0 | 15.8 | 0 | 1.17 | 12.73 | 1.98 | 84.2 | 0 | -0.22749 | 1.058147 | 0.641359 | 26.61247 | -5.41654 | 2.007514 |
| ANALOC-E_60 | 8 | Campolina | Rocky Mt. rack | Marcha Batida | M | 44.4 | 0 | 0 | 43.4 | 0 | 0 | 0 | 12.2 | 0 | 1.13 | 13.2 | 2.02 | 87.8 | 0 | 0.153243 | 0.976255 | 0.952905 | 25.85938 | -9.71468 | 2.62618 |
| ANALOC-E_31 | 5 | Campolina | Rocky Mt. rack | Marcha Batida | M | 49 | 0 | 0 | 33 | 0 | 0 | 0 | 18 | 0 | 1.2 | 12.76 | 1.97 | 82 | 0 | -0.31296 | 0.704589 | 0.558906 | 16.62237 | -1.004 | 1.328447 |
| ANALOC-E_25 | 5 | Campolina | Fox Trot | Marcha Batida | M | 52.9 | 0 | 0 | 33.7 | 0 | 0 | 0 | 13.5 | 0 | 1.14 | 12.66 | 2.03 | 86.6 | 0 | 0.023235 | 0.419219 | 0.644503 | 13.44459 | -6.02891 | 2.085585 |
| ANALOC-E_28 | 5 | Campolina | Rocky Mt. rack | Marcha Batida | M | 51 | 0 | 0 | 28.1 | 0 | 0 | 0 | 20.8 | 0 | 1.23 | 13.9 | 2.06 | 79.1 | 0 | 0.093029 | 0.582319 | 1.220059 | 12.40797 | 3.193745 | 0.660788 |
| ANALOC-E_26 | 5 | Campolina | Fox Trot | Marcha Batida | M | 55.4 | 0 | 0 | 7.9 | 0 | 0 | 0 | 36.6 | 0 | 1.45 | 11.99 | 1.87 | 63.3 | 0 | -1.52656 | 0.289778 | -0.21612 | -1.42534 | 24.98669 | -2.54809 |
| ANALOC-E_42 | 5 | Campolina | Unclassified | Marcha Picada | M | 37.6 | 0 | 0 | 46.8 | 0 | 0 | 0 | 15.6 | 0 | 1.17 | 11.73 | 1.97 | 84.4 | 0 | -0.51555 | 1.237417 | 0.213733 | 33.67686 | -6.86973 | 2.24656 |
| ANALOC-E_37 | 6 | Campolina | Sobreandando | Marcha Picada | M | 29.7 | 0 | 0 | 41.8 | 0 | 0 | 0 | 28.6 | 0 | 1.33 | 12.03 | 1.69 | 71.5 | 0 | -1.74424 | 2.032904 | -0.27824 | 38.40973 | 8.272836 | 0.118702 |
| ANALOC-E_50 | 6 | Campolina | Coon Rack | Marcha Picada | M | 29.1 | 0 | 0 | 55.3 | 0 | 0 | 0 | 15.5 | 0 | 1.17 | 11.98 | 1.9 | 84.4 | 0 | -0.6633 | 1.836967 | 0.25524 | 45.50666 | -8.97991 | 2.556854 |
| ANALOC-E_23 | 8 | Campolina | Coon Rack | Marcha Picada | M | 29.1 | 0 | 0 | 55.3 | 0 | 0 | 0 | 15.5 | 0 | 1.17 | 11.98 | 1.9 | 84.4 | 0 | -0.6633 | 1.836967 | 0.25524 | 45.50666 | -8.97991 | 2.556854 |
| ANALOC-E_19 | 6 | Criollo | Unclassified | Broken Pace | M | 0 | 0 | 0 | 78.9 | 0 | 6.4 | 5.5 | 0 | 9.2 | 0.74 | 12.1 | 2.04 | 78.9 | 11.9 | 6.57569 | 5.675142 | -4.71827 | 80.53449 | -27.867 | -11.8331 |
| ANALOC-E_45 | 8 | Criollo | Broken Trot | Broken Trot | M | 87 | 0 | 0 | 0 | 0 | 3 | 6 | 0 | 4 | 0.84 | 12.94 | 2.21 | 87 | 9 | 5.304522 | -0.3692 | -1.91224 | -35.6532 | -10.4268 | -7.99009 |
| ANALOC-E_18 | 6 | Criollo | Broken Trot | Broken Trot | M | 83.3 | 0 | 0 | 0 | 0 | 0 | 3.9 | 0 | 12.7 | 0.74 | 14.34 | 2.26 | 83.3 | 3.9 | 5.541984 | -0.62811 | -1.22425 | -32.8133 | -9.90014 | -13.1729 |
| ANALOC-E_22 | 5 | Mangalarga | Toelt | Marcha Centro | M | 40 | 0 | 0 | 40 | 0 | 0 | 0 | 20 | 0 | 1.22 | 10.97 | 1.78 | 80 | 0 | -1.37115 | 1.252569 | -0.58605 | 28.18777 | -0.5211 | 1.305805 |
| ANALOC-E_41 | 5 | Mangalarga | Unclassified | Marcha Trotada | F | 82.6 | 0 | 0 | 4.6 | 1.8 | 0 | 0 | 11 | 0 | 1.16 | 11.7 | 1.97 | 87.2 | 0 | -0.18343 | -2.22273 | -0.83768 | -27.917 | -1.37758 | 0.408536 |
| ANALOC-E_66 | 8 | Mangalarga | Unclassified | Marcha Trotada | F | 76.8 | 0 | 0 | 1.8 | 0.9 | 0 | 0 | 20.5 | 0 | 1.25 | 10.52 | 1.82 | 78.6 | 0 | -1.27175 | -1.40004 | -1.37448 | -23.8994 | 9.306455 | -0.72447 |
| ANALOC-E_57 | 5 | Mangalarga | Unclassified | Marcha Trotada | F | 80.5 | 0 | 0 | 6.2 | 0 | 0 | 0 | 13.3 | 0 | 1.14 | 10.52 | 1.83 | 86.7 | 0 | -0.90538 | -1.10243 | -0.98191 | -24.9778 | 0.325724 | 0.966286 |
| ANALOC-E_64 | 5 | Mangalarga | Unclassified | Marcha Trotada | F | 87.1 | 0 | 0 | 6.9 | 0 | 0 | 0 | 5.2 | 0 | 1.04 | 12.15 | 2.17 | 94 | 0 | 0.747262 | -1.73155 | 0.472552 | -30.7266 | -8.37681 | 1.855748 |
| ANALOC-E_58 | 5 | Mangalarga | Unclassified | Marcha Trotada | F | 85 | 0 | 0 | 5 | 0 | 0 | 0 | 0 | 0 | 0.9 | 12.58 | 2.33 | 90 | 0 | 1.476419 | -2.02924 | 1.003158 | -31.0296 | -11.5519 | -1.66427 |
| ANALOC-E_2 | 5 | Mangalarga | Unclassified | Marcha Trotada | F | 76.7 | 0 | 0 | 9.5 | 0 | 0 | 0 | 13.8 | 0 | 1.15 | 10.86 | 1.94 | 86.2 | 0 | -0.57434 | -1.0043 | -0.56938 | -19.9267 | 0.077364 | 1.022831 |
| ANALOC-E_59 | 5 | Mangalarga | Unclassified | Marcha Trotada | F | 91.3 | 0 | 0 | 1 | 0 | 1.9 | 3.9 | 1.9 | 0 | 0.96 | 11.81 | 1.88 | 92.3 | 5.8 | 2.163191 | -0.85175 | -1.58998 | -38.0832 | -9.76141 | -1.84062 |
| ANALOC-E_10 | 5 | Mangalarga | Unclassified | Marcha Trotada | F | 88.2 | 0 | 0 | 6.4 | 0 | 0 | 0 | 2.7 | 0 | 1 | 12.02 | 2.04 | 94.6 | 0 | 0.490943 | -1.6719 | 0.141496 | -32.231 | -10.5398 | 1.340929 |
| ANALOC-E_11 | 5 | Mangalarga | Unclassified | Marcha Trotada | F | 82.2 | 0 | 0 | 0 | 2.8 | 0 | 0 | 15 | 0 | 1.23 | 11.7 | 1.93 | 82.2 | 0 | -0.4797 | -2.6998 | -1.33207 | -29.9398 | 4.232742 | -0.85135 |
| ANALOC-E_34 | 5 | Mangalarga | Unclassified | Marcha Trotada | F | 80.9 | 0 | 0 | 1.8 | 0 | 0 | 0 | 17.3 | 0 | 1.19 | 11.31 | 1.92 | 82.7 | 0 | -0.64452 | -1.17915 | -0.47476 | -27.4851 | 5.672925 | 0.182563 |
| ANALOC-E_67 | 5 | Mangalarga | Unclassified | Marcha Trotada | F | 88.3 | 0 | 0 | 1 | 1 | 0 | 0 | 7.8 | 0 | 1.08 | 12.17 | 1.93 | 89.3 | 0 | 0.007868 | -2.04541 | -0.43984 | -34.9855 | -3.72718 | 0.250064 |
| ANALOC-E_40 | 8 | Mangalarga | Unclassified | Marcha Trotada | M | 94.1 | 0 | 0 | 0 | 0 | 0 | 0 | 5.9 | 0 | 1.06 | 11.41 | 2.08 | 94.1 | 0 | 0.329717 | -2.06581 | -0.11218 | -40.3202 | -6.15196 | 1.840675 |
| ANALOC-E_30 | 5 | Mangalarga | Unclassified | Marcha Trotada | M | 88.2 | 0 | 0 | 4.2 | 0 | 0 | 0 | 2.5 | 0 | 0.99 | 11.27 | 2.07 | 92.4 | 0 | 0.388194 | -1.87035 | -0.14937 | -33.6372 | -9.7191 | 0.178722 |
| ANALOC-E_20 | 5 | Mangalarga | Unclassified | Marcha Trotada | M | 87.5 | 0 | 0 | 4.8 | 0 | 0 | 0 | 7.6 | 0 | 1.08 | 12.01 | 1.93 | 92.3 | 0 | -0.00463 | -1.43179 | -0.11174 | -32.0153 | -5.44634 | 1.729265 |
| ANALOC-E_1 | 5 | Mangalarga | Unclassified | Marcha Trotada | M | 85.6 | 0 | 0 | 3.6 | 0 | 0 | 0 | 10.8 | 0 | 1.11 | 11.72 | 2.01 | 89.2 | 0 | -0.01534 | -1.48364 | -0.08618 | -30.8653 | -1.74234 | 1.241405 |
| ANALOC-E_54 | 5 | Mangalarga | Unclassified | Marcha Trotada | M | 88 | 0 | 0 | 0.9 | 0 | 0 | 0 | 9.3 | 0 | 1.14 | 11.17 | 1.86 | 88.9 | 0 | -0.46537 | -1.52049 | -0.66836 | -34.6059 | -2.32789 | 0.532521 |
| ANALOC-E_33 | 8 | Mangalarga Marchador | Rocky Mt. rack | Marcha Batida | F | 41.7 | 0 | 0 | 22.3 | 0 | 0 | 0 | 35.9 | 0 | 1.44 | 12.64 | 2.01 | 64 | 0 | -1.03028 | 0.944074 | 0.496407 | 18.00518 | 20.80536 | -1.86394 |
| ANALOC-E_14 | 10 | Mangalarga Marchador | Fox Trot | Marcha Batida | F | 68.8 | 0 | 0 | 19.8 | 0 | 0 | 0 | 21 | 0 | 1.23 | 12.15 | 1.97 | 88.6 | 0 | -0.51025 | -0.20749 | 0.14779 | -6.41423 | 3.316684 | 4.760433 |
| ANALOC-E_55 | 8 | Mangalarga Marchador | Fox Trot | Marcha Batida | F | 59.4 | 0 | 0 | 17.8 | 0 | 0 | 0 | 22.8 | 0 | 1.26 | 12.36 | 1.93 | 77.2 | 0 | -0.67728 | 0.104078 | 0.161737 | -0.20874 | 7.77479 | -0.01014 |
| ANALOC-E_3 | 6 | Mangalarga Marchador | Unclassified | Marcha Batida | F | 60.4 | 0 | 0 | 21.9 | 0 | 0 | 0 | 17.7 | 0 | 1.19 | 13.9 | 2.06 | 82.3 | 0 | 0.263106 | 0.075234 | 1.16523 | 0.884119 | 1.316669 | 0.928512 |
| ANALOC-E_29 | 5 | Mangalarga Marchador | Unclassified | Marcha Batida | F | 63.4 | 0 | 0 | 20.4 | 0 | 0 | 0 | 16.1 | 0 | 1.18 | 13.12 | 1.88 | 83.8 | 0 | -0.31456 | 0.070453 | 0.423347 | -2.57867 | -0.02462 | 1.065158 |
| ANALOC-E_48 | 8 | Mangalarga Marchador | Fox Trot | Marcha Batida | F | 46.7 | 0 | 0 | 14.4 | 0 | 0 | 0 | 38.9 | 0 | 1.38 | 12.44 | 1.73 | 61.1 | 0 | -1.90679 | 1.013018 | -0.24809 | 9.638456 | 25.88978 | -2.59033 |
| ANALOC-E_24 | 5 | Mangalarga Marchador | Rocky Mt. rack | Marcha Batida | F | 43.8 | 0 | 0 | 29.2 | 0 | 0 | 0 | 13.6 | 0 | 1.31 | 12.31 | 1.82 | 73 | 0 | -0.64824 | 0.77681 | 0.036053 | 17.61731 | -1.97115 | -4.32172 |
| ANALOC-E_32 | 5 | Mangalarga Marchador | Rocky Mt. rack | Marcha Batida | F | 41.6 | 0 | 0 | 36.6 | 0 | 0 | 0 | 21.8 | 0 | 1.24 | 11.58 | 1.8 | 78.2 | 0 | -1.2369 | 1.196185 | -0.29799 | 25.08087 | 2.223274 | 0.896141 |
| ANALOC-E_5 | 8 | Mangalarga Marchador | Fox Trot | Marcha Batida | F | 58.8 | 0 | 0 | 19.6 | 0 | 0 | 0 | 21.6 | 0 | 1.24 | 11.44 | 1.8 | 78.4 | 0 | -1.19321 | 0.216775 | -0.51027 | 1.212286 | 6.056333 | 0.244245 |
| ANALOC-E_16 | 4 | Mangalarga Marchador | Unclassified | Marcha Batida | F | 83 | 0 | 0 | 0 | 0 | 0 | 0 | 17 | 0 | 1.18 | 10.7 | 2.09 | 83 | 0 | -0.33916 | -1.60022 | -0.41132 | -30.265 | 5.779079 | 0.15586 |
| ANALOC-E_52 | 5 | Mangalarga Marchador | Rocky Mt. rack | Marcha Batida | F | 49 | 0 | 0 | 28.8 | 0 | 0 | 0 | 22.1 | 0 | 1.25 | 12.13 | 1.95 | 77.8 | 0 | -0.69627 | 0.63376 | 0.196005 | 14.56098 | 4.424391 | 0.49156 |
| ANALOC-E_56 | 5 | Mangalarga Marchador | Unclassified | Marcha Batida | F | 82 | 0 | 0 | 3 | 0 | 0 | 0 | 15 | 0 | 1.16 | 11.4 | 2.06 | 85 | 0 | -0.16008 | -1.41076 | -0.13131 | -27.8966 | 2.914983 | 0.579828 |
| ANALOC-E_6 | 6 | Mangalarga Marchador | Fox Trot | Marcha Batida | F | 56.1 | 0 | 0 | 24.5 | 0 | 0 | 0 | 19.4 | 0 | 1.21 | 14.35 | 2.17 | 80.6 | 0 | 0.568683 | 0.207593 | 1.621487 | 6.046896 | 2.524836 | 0.774822 |
| ANALOC-E_49 | 8 | Mangalarga Marchador | Fox Trot | Marcha Batida | F | 67.6 | 0 | 0 | 11.8 | 0 | 0 | 0 | 20.6 | 0 | 1.23 | 12.64 | 1.99 | 79.4 | 0 | -0.32606 | -0.39384 | 0.36093 | -10.5619 | 6.838796 | 0.082997 |
| ANALOC-E_68 | 10 | Mangalarga Marchador | Fox Trot | Marcha Batida | F | 55.8 | 0 | 0 | 12.5 | 0 | 0 | 0 | 31.7 | 0 | 1.38 | 12.87 | 2.07 | 68.3 | 0 | -0.58252 | 0.123481 | 0.639291 | 0.468701 | 18.60316 | -1.57373 |
| ANALOC-E_44 | 5 | Mangalarga Marchador | Coon Rack | Marcha Picada | F | 39 | 0 | 0 | 50 | 0 | 0 | 0 | 11 | 0 | 1.12 | 11.96 | 1.87 | 89 | 0 | -0.51382 | 1.34852 | 0.136443 | 33.96861 | -12.5761 | 3.073196 |
| ANALOC-E_4 | 10 | Mangalarga Marchador | Unclassified | Unclassified Gait | F | 91.6 | 0 | 0.9 | 0.9 | 0 | 0 | 4.7 | 1.9 | 0 | 0.97 | 13.25 | 2.19 | 93.4 | 4.7 | 3.617377 | -1.30278 | 2.325884 | -38.3868 | -9.77408 | -1.67483 |
| ANALOC-E_47 | 6 | Mangalarga Marchador | Unclassified | Unclassified Gait | F | 96 | 1 | 0 | 0 | 0 | 0 | 0 | 3 | 0 | 1.03 | 10.68 | 2.12 | 97 | 0 | 0.570131 | -3.90233 | -1.60404 | -42.1812 | -9.05706 | 1.823961 |
| ANALOC-E_17 | 5 | Mangalarga Marchador | Unclassified | Unclassified Gait | F | 80.4 | 0 | 0 | 0.9 | 0.9 | 0 | 3.7 | 14 | 0 | 1.13 | 13.67 | 2.26 | 81.3 | 3.7 | 2.1712 | -1.59136 | 0.706209 | -28.1218 | 3.112612 | -2.76812 |
| ANALOC-E_13 | 6 | Mangalarga Marchador | Marcha Batida | Unclassified Gait | F | 97 | 0 | 1 | 0 | 0 | 0 | 0 | 2 | 0 | 1.02 | 12.16 | 2.27 | 98 | 0 | 2.0928 | -2.09209 | 2.503869 | -43.0852 | -10.1396 | 1.9728 |
| ANALOC-E_35 | 6 | Mangalarga Marchador | Rocky Mt. rack | Marcha Batida | M | 45.2 | 0 | 0 | 36.5 | 0 | 0 | 0 | 17.7 | 0 | 1.19 | 13.12 | 1.73 | 81.7 | 0 | -0.84239 | 1.273912 | 0.252247 | 21.68753 | -2.03193 | 1.238379 |
| ANALOC-E_15 | 6 | Mangalarga Marchador | Fox Trot | Marcha Batida | M | 47.4 | 0 | 0 | 22.7 | 0 | 0 | 0 | 29.9 | 0 | 1.35 | 11.32 | 1.69 | 70.1 | 0 | -1.90497 | 0.948229 | -0.76636 | 13.05056 | 14.23957 | -0.89116 |
| ANALOC-E_61 | 12 | Mangalarga Marchador | Rocky Mt. rack | Marcha Batida | M | 40.9 | 0 | 0 | 32.3 | 0 | 0 | 0 | 26.9 | 0 | 1.31 | 11.32 | 1.63 | 73.2 | 0 | -1.95827 | 1.414673 | -0.80809 | 23.63266 | 8.707695 | -0.00452 |
| ANALOC-E_65 | 6 | Mangalarga Marchador | Fox Trot | Marcha Batida | M | 56.4 | 0 | 0 | 14.9 | 0 | 0 | 0 | 28.7 | 0 | 1.34 | 12.63 | 1.83 | 71.3 | 0 | -1.12944 | 0.409143 | 0.050123 | 1.095164 | 14.80706 | -1.02205 |
| ANALOC-E_38 | 6 | Mangalarga Marchador | Unclassified | Marcha Batida | M | 90 | 0 | 0 | 0 | 0 | 0 | 0 | 10 | 0 | 1.19 | 11.7 | 1.84 | 90 | 0 | -0.40521 | -1.494 | -0.48197 | -36.6061 | -1.745 | 1.218356 |
| ANALOC-E_39 | 4 | Mangalarga Marchador | Unclassified | Marcha Batida | M | 65 | 0 | 0 | 17 | 0 | 0 | 0 | 18 | 0 | 1.19 | 11.7 | 1.84 | 82 | 0 | -0.8471 | -0.13613 | -0.33225 | -5.67166 | 2.805916 | 0.686327 |
| ANALOC-E_7 | 4 | Mangalarga Marchador | Rocky Mt. rack | Marcha Batida | M | 40.4 | 0 | 0 | 36.5 | 0 | 0 | 0 | 23.1 | 0 | 1.26 | 11.01 | 1.77 | 76.9 | 0 | -1.51685 | 1.231629 | -0.61921 | 26.11918 | 3.644416 | 0.694807 |
| ANALOC-E_8 | 6 | Mangalarga Marchador | Unclassified | Marcha Picada | M | 23 | 0 | 0 | 34 | 0 | 0 | 0 | 43 | 0 | 1.55 | 10.27 | 1.58 | 57 | 0 | -3.10661 | 2.274476 | -1.37075 | 40.66276 | 25.6296 | -2.42605 |
